# Hepatocellular Carcinoma Surveillance: Benefit of Serum Alfa-fetoprotein in Real-world Practice

**DOI:** 10.5005/jp-journals-10018-1268

**Published:** 2018-05-01

**Authors:** Patharapan Lersritwimanmaen, Supot Nimanong

**Affiliations:** 1 Division of Gastroenterology, Department of Medicine, Faculty of Medicine, Siriraj Hospital, Mahidol University, Bangkok, Thailand

**Keywords:** Alfa-fetoprotein, Hepatocellular carcinoma, Surveillance.

## Abstract

**Introduction:**

Better treatment outcome of early-stage hepatocellular carcinoma (HCC) warrants employment of screening programs, in which ultrasonography (US) and serum alfa-fetoprotein (AFP) have been recommended. Considering cost-effectiveness, serum AFP has recently been withdrawn from several guidelines for HCC surveillance. However, there were limited studies on benefits of AFP for HCC surveillance in Thailand.

**Materials and methods:**

This is a retrospective study of a proportion of HCC cases in which a diagnostic study was triggered by high serum AFP levels, but US failed to detect the lesion. Patients who received diagnostic imaging for HCC at Siriraj Hospital between January 1, 2012 and December 31, 2014 were included. All the patients must fulfill criteria for HCC surveillance according to American Association for the Study of Liver Diseases (AASLD) practice guidelines on the management of HCC 2010 or European Association for the Study of the Liver-European Organisation for Research and Treatment of Cancer (EASL-EORTC) Clinical Practice Guidelines: Management of HCC 2012. Previous diagnosis of any liver malignancy was excluded. Demographic data, underlying liver diseases, screening of AFP and US results, and definite diagnosis of HCC were recorded.

**Results:**

Of the 452 cases who fulfilled inclusion and exclusion criteria, chronic hepatitis B, C, and alcoholic cirrhosis were accountable for 53.8, 25.9, and 7.3% respectively. Totally, 150 cases were diagnosed with HCC. Additional HCC detection rate by high serum AFP but failed US of 15.3% was demonstrated. Subgroup analysis revealed significant benefit of AFP in cirrhotic patients with chronic hepatitis B and C (p-value 0.004 and 0.002). No significant benefit was observed in cirrhosis of other causes and in noncirrhotic chronic hepatitis B.

**Conclusion:**

We reported a 15.3% additional benefit of serum AFP for HCC surveillance in conjunction with US of liver. Chronic hepatitis B and C with cirrhosis significantly derived the benefit from serum AFP screening.

**How to cite this article:** Lersritwimanmaen P, Nimanong S. Hepatocellular Carcinoma Surveillance: Benefit of Serum Alfa-fetoprotein in Real-world Practice. Euroasian J Hepato-Gastroenterol 2018;8(1):83-87.

## INTRODUCTION

Hepatocellular carcinoma is one of the most common malignancies. It is the second and sixth leading cause of cancer-related deaths worldwide in men and women respectively.^1^ Without extensive surveillance programs, most of the patients are diagnosed in intermediate to advanced stage. About 60 and 30% of patients will survive after 1 year of diagnosis in intermediate and advanced stages respectively. Early diagnosis results in 1-year survival rate of more than 80%.^2^

Ultrasonography is recommended by all current standard guidelines for the management of HCC. Pooled sensitivity of 94% has been reported.^3^ The sensitivity profile of US for HCC surveillance varies due to operator dependency and its use is limited by the location of tumor and characters of the patient, including obesity, chest wall deformity, and advanced cirrhotic liver. Additional methods have been proposed to increase HCC detection rate. Of those, serum AFP level is most frequently evaluated, but the benefit is controversial. Combined with US, serum AFP provides additional detection of 6 to 8%. Current American and European guidelines, AASLD, and EASL-EORTC guidelines do not recommend serum AFP for HCC surveillance due to cost-benefit issues. However, Japan Society of Hepatology (JSH) and Asian Pacific Association for the Study of the Liver (APASL) guidelines still recommend serum AFP for HCC surveillance.^4^

Information in Thai population, in which proportions of the underlying causes of HCC differ from those of western regions, is limited. Therefore, we aimed to figure out the number of patients potentially benefited from serum AFP for HCC surveillance, and determine the subgroups that would get most benefit.

## MATERIALS AND METHODS

The study protocol was approved by the Institutional Review Board of Faculty of Medicine Siriraj Hospital, Mahidol University. We conducted a retrospective analysis of the results of triple-phase computed tomography (CT) and magnetic resonance imaging (MRI) of liver performed at the Department of Radiology, Faculty of medicine Siriraj Hospital, between January 1, 2012 and December 31, 2014. In the case of multiple studies, the first study in this period was selected. We included all the patients who had documented US of liver and serum AFP level in the period of 6 months prior to CT or MRI liver, and fulfilled the indications for HCC surveillance according to AASLD practice guidelines on the management of HCC update 2010 and EASL-EORTC Clinical Practice Guidelines: Management of HCC 2012.^5,6^ In brief, patients with cirrhosis of any causes, male hepatitis B carriers over age 40, female hepatitis B carriers over age 50, hepatitis B carriers with family history of HCC, and patients with chronic hepatitis C with F3 liver fibrosis according to METAVIR score were included. The inclusion criterion of follow-up period of 2 years by US, CT, or MRI of liver was additionally applied to all the patients diagnosed negative for HCC by the first diagnostic imaging. We excluded the patients with previous diagnosis of any liver malignancy and those with other indications for liver imaging.

The demographic data, underlying liver diseases, results of the most recent serum AFP level and US of liver, and final diagnosis of HCC were recorded. Positive screening US was defined as liver nodules, including new lesions or increasing size of the previously described lesions. Positive serum AFP was defined as the level more than 20 IU/mL. The diagnosis of HCC was established by histology or imaging criteria by CT or MRI liver. Suspected HCCs from the screening US or serum AFP level were diagnosed free of HCC when the follow-up period of 2 years did not result in definite diagnosis of HCC.

### Statistical Analysis

All statistical analyses were performed using the Statistical Program for Social Sciences (version 18.0; SPSS Inc., Chicago, Illinois, USA). Patient characteristics were described using descriptive statistics, including frequencies and percentage for categorical variables. Continuous variables were reported as mean ± standard deviation (SD). Student’s t-test was performed to test difference between continuous variables. Z-test was used for difference between proportions. The association between categorical variables and final diagnosis of HCC was assessed by chi-squared test or Fischer exact test. The p-value less than 0.05 was considered statistically significant.

## RESULTS

### Baseline Characteristics

The records of 10,606 CT and MRI liver during January 1, 2012 to December 31, 2014 from 4,615 patients were obtained from the Department of Radiology. After International Classification of Diseases 10th revision and medical records were reviewed, 452 patients who fulfilled the inclusion and exclusion criteria were eligible for further analysis. The baseline characteristics were outlined in Table 1. Of the total 452 patients, 150 were diagnosed with HCC within 2 years since the index imaging study. Overall, mean age was 59.2 ± 9.8 years, with significantly older patients in HCC cases (62.4 ± 10.2 years). Majority of the patients were male (61.7%). Baseline cirrhotic livers were 77.4% of total number, with higher frequency found in HCC cases (90%) comparing with non-HCC cases (71.2%).

Among the underlying liver diseases, chronic viral hepatitis B was most prevalent (53.8%). Chronic viral hepatitis C (25.9%) and alcoholic cirrhosis (7.3%) were the second and third most common respectively. Other identified conditions were accountable for less than 10% of the patients, and the rest (4%) were documented cryptogenic cirrhosis.

Screening US of liver revealed suspicious features in 353 patients (78.1%). In HCC group, 81.3% of cases were US positive. The positive rate was also high in non-HCC group (76.5%). Screening serum AFP was positive in 18.1, 36, and 9.3% of overall patients, HCC, and non-HCC group respectively.

### Combination of US and Serum AFP Level and HCC Detection Rate

Combinations of US and serum AFP level are shown in Table 2. From a total of 452 patients, 9.3, 68.8, 8.8, and 13.1% were dual positive, US positive alone, AFP positive alone, and dual negative respectively. Proportion of patients with dual positive screening test was more in the group with final diagnosis of HCC (20.6 *vs* 3.6%). The same manner was observed in AFP positive alone (15.3 *vs* 5.6%).

**Table 1:** **Table 1:** Baseline characteristics between patients with and without diagnosis of HCC

		*Total (n = 452)*		*HCC (n = 150)*		*Non-HCC (n = 302)*		*p-value*	
Age (years), mean ± SD		59.2 ± 9.8		62.4 ± 10.2		57.6 ± 9.2		<0.001	
Male sex, n (%)		279 (61.7)		90 (60)		189 (62.6)		0.595	
Cirrhosis, n (%)		350 (77.4)		135 (90)		215 (71.2)		<0.001	
*Underlying liver disease, n (%)*									
• Chronic hepatitis B (CHB)		243 (53.8)		63 (42)		180 (59.6)			
• Chronic hepatitis C (CHC)		117 (25.9)		53 (35.3)		64 (21.2)			
• CHB/CHC coinfection		7 (1.5)		4 (2.7)		3 (1)			
• Alcoholic cirrhosis		33 (7.3)		8 (5.3)		25 (8.3)			
• CHB/alcoholic		3 (0.7)		1 (0.7)		2 (0.7)			
• CHC/alcoholic		4 (0.9)		3 (2)		1 (0.3)			
• CHB/CHC/alcoholic		1 (0.2)		1 (0.7)		0			
• Nonalcoholic steatohepatitis		16 (3.5)		4 (2.7)		12 (4)			
• Autoimmune hepatitis		3 (0.7)		2 (1.3)		1 (0.3)			
• Primary biliary cholangitis		3 (0.7)		2 (1.3)		1 (0.3)			
• Wilson disease		2 (0.4)		0		2 (0.7)			
• Hemochromatosis		1 (0.2)		1 (0.7)		0			
• Methotrexate-induced cirrhosis		1 (0.2)		1 (0.7)		0			
• Cryptogenic cirrhosis		18 (4)		7 (4.7)		11 (3.6)			
Screening US positive, n (%)		353 (78.1)		122 (81.3)		231 (76.5)			
Screening AFP positive (>20 lU/mL), n (%)		82 (18.1)		54 (36)		28 (9.3)		<0.001	

**Table 2:** **Table 2:** Screening results

				*With HCC, n (%)*		*Without HCC, n (%)*	
US positive		AFP positive		31 (20.6)		11 (3.6)	
US positive		AFP negative		91 (60.6)		220 (72.8)	
US negative		AFP positive		23 (15.3)		17 (5.6)	
US negative		AFP negative		5 (3.3)		54 (17.9)	
		Total		150		302	

**Table 3:** **Table 3:** Hepatocellular carcinoma detection rate

		*HCC detection rate (%)*		*False positive rate (%)*	
Single test US positive		81.3		76.5	
Single test AFP positive		36.0		9.3	
Combined US positive and/or AFP positive		96.7		82.1	

*Overestimated due to study limitation

**Table 4:** **Table 4:** Subgroup analysis of additional benefit of serum AFP for HCC detection

				*HCC detection (%)*									
*With HCC*				*US positive*		*US and/or AFP positive*		p-value*		*ADR (%)*		*NND*	
Total (452)		150		122 (81.3)		145 (96.7)		<0.001		15.3		20	
CHB** (254)		69		54 (78.3)		66 (95.7)		0.002		17.4		22	
• With cirrhosis (154)		54		40 (74.1)		51 (94.4)		0.004		20.4		14	
• No cirrhosis (100)		15		14 (93.3)		15 (100)		0.308		6.7		100	
CHC*** (129)		61		50 (82.0)		60 (98.4)		0.002		16.4		13	
Alcoholic cirrhosis (33)		8		7 (87.5)		8 (100)		0.302		12.5		33	
Cryptogenic cirrhosis (18)		7		5 (71.4)		6 (85.7)		0.515		14.3		18	

*Z-test comparing proportion of US positive *vs* US and/or AFP positive; **including CHB/CHC coinfection and CHB with alcoholic cirrhosis; ***including CHC with alcoholic cirrhosis; CHB: Chronic hepatitis B; CHC: Chronic hepatitis C; ADR: Additional detection rate

The HCC detection rate was calculated for each single screening test and combined tests, as shown in Table 3. With US, 81.3% of HCC cases were detected, while only 36% of HCC cases were detected by serum AFP. Detection rate increased to 96.7% with combination of US and serum AFP.

### Subgroup Analysis of Additional Benefits of Serum AFP for HCC Detection

The HCC detection rate in patient subgroups is demonstrated in Table 4. Overall, 15.3% of HCC patients gained additional benefit from combined screening tests with statistical significance. The benefit was observed in patients with underlying chronic hepatitis B and C, but not in alcoholic and cryptogenic cirrhosis. Since chronic hepatitis B patients enrolled in the surveillance program included significant number of noncirrhotic cases, subgroup analysis of chronic hepatitis B with cirrhosis and without cirrhosis was performed. Significant different HCC detection rates were observed in chronic hepatitis B with cirrhosis, but not in without cirrhosis group. Number of serum AFP tests needed to be done to detect one additional HCC case (NND) was calculated. However, due to study design, NND in this study might not reflect the whole population.

## DISCUSSION

In this study, we found additional benefit of serum AFP for HCC surveillance. Using serum AFP cutoff level over 20 IU/mL in combination with US of liver, 15.3% more HCC cases were detected. The difference in proportion of HCC cases detected by US alone *vs* combined US and serum AFP reached statistical significance. By contrast, previous meta-analysis has reported no significant additional benefit of serum AFP in conjunction with US.^3^ The majority of studies included in that meta-analysis were conducted in European countries. Only two studies were conducted in Japan; one of them reported 12% additional benefit of serum AFP. Since chronic viral hepatitis B was more prevalent in Asian countries, the difference in prevalence of underlying liver diseases might influence the results.

Additional benefit of serum AFP was demonstrated in HCC cases that US failed to detect the lesion, but serum AFP was positive. Since the inclusion criteria include cases in which serum AFP and US were performed within 6 months prior to the index diagnostic studies, interval between the date of US and serum AFP might interfere with the interpretation. To exclude this possibility, we thoroughly reviewed the 23 cases of HCC with US negative and serum AFP positive. Characteristics of 23 cases of HCC with US negative/AFP positive are shown in Table 5. Serum AFP and US of liver were performed within 2 months apart in 18 patients. Only in 5 patients, the tests were performed within 2 to 6 months.

Subgroup analysis revealed that additional benefit of serum AFP was significant in patients with cirrhosis from chronic hepatitis B and C. Association of high serum AFP level in HCC patients with hepatitis B infection has been reported.^7^ However, our results showed the benefit of serum AFP was only in cirrhotic chronic hepatitis B patients, but not in noncirrhotic cases. This finding might be explained by limitation of US to detect abnormal lesions in cirrhotic liver.

There are several advantages of this retrospective study. First, since US is operator dependence, sensitivity and specificity of US for HCC surveillance in a controlled study with specified radiologists might be higher than average. The sensitivity of 81.3% derived from this study represents real practice in Siriraj Hospital, and probably in Thailand. Second, we started from patients who underwent diagnostic imaging for HCC, and subsequently reviewed screening test results. This approach allowed us to retrieve more number of HCC cases.

For limitations, false positive rate and NND HCC were subject to error, since majority of patients with US negative and AFP negative had not been scheduled for diagnostic imaging. Second, although most patients received regular US every 6 months for HCC surveillance, they did not receive regular serum AFP monitoring. Therefore, the results might be interfered by the interval between screening tests to diagnostic imaging and there might be some differences of the characteristics between the patients who received AFP for HCC surveillance or not.

**Table 5:** **Table 5:** Characteristics of 23 cases of HCC with US negative/AFP positive

*Patient no.*		*Age (years)*		*Sex (M/F)*		*Cirrhosis (Y/N)*		*Underlying liver diseases*		*AFP level (IU/mL)*		*Interval between US and AFP (days)*	
1		*62*		F		Y		CHC		450.5		0	
2		64		M		Y		CHB		56.8		0	
3		82		M		Y		Alcohol		131.5		0	
4		71		F		N		CHB		78.4		18	
5		58		F		Y		CHB		2320.0		9	
6		69		M		Y		CHB		282.6		16	
7		69		M		Y		Cryptogenic		47.8		0	
8		36		M		Y		CHB		226.0		0	
9		75		F		Y		CHB		53.0		66	
10		82		M		Y		CHB		235.1		17	
11		57		F		Y		CHC		74.7		2	
12		61		M		Y		CHC		33.5		104	
13		74		F		Y		CHC		23.8		0	
14		58		M		Y		CHC		21.4		6	
15		58		F		Y		CHB/CHC		73.0		171	
16*		62		F		Y		CHC		137.1		14	
17		64		M		Y		CHB		40.3		118	
18		51		M		Y		CHC		849.4		2	
19		57		F		Y		CHC		67.5		17	
20		38		M		Y		CHC/Alcohol		55.4		135	
21**		57		M		Y		CHB		26.3		34	
22		69		M		Y		CHB		420.0		30	
23		57		F		Y		CHB		24.9		25	

*HCC was diagnosed 17 months after the index diagnostic study; **HCC was diagnosed 23 months after the index diagnostic study; CHB: Chronic hepatitis B; CHC: Chronic hepatitis C

## CONCLUSION

We reported significant additional benefit of serum AFP level for HCC surveillance in conjunction with US of liver. The benefit is pronounced in cirrhotic patients from chronic hepatitis B and C. We propose that serum AFP screening should be done selectively. More studies are needed to establish the recommendations.
